# Plasma thioredoxin levels during post-cardiac arrest syndrome: relationship with severity and outcome

**DOI:** 10.1186/cc12492

**Published:** 2013-01-29

**Authors:** Nicolas Mongardon, Virginie Lemiale, Didier Borderie, Anne Burke-Gaffney, Sébastien Perbet, Nathalie Marin, Julien Charpentier, Frédéric Pène, Jean-Daniel Chiche, Jean-Paul Mira, Alain Cariou

**Affiliations:** 1 Medical Intensive Care Unit, Cochin Hospital, Hôpitaux Universitaires Paris Centre, Assistance Publique des Hôpitaux de Paris, 27 rue du Faubourg Saint-Jacques, 75014 Paris, France; 2 Université Paris Descartes, Sorbonne Paris Cité, Faculté de Médecine, 15 rue de l'Ecole de Médecine, 75006 Paris, France; 3 Department of Biochemistry, Cochin Hospital, Hôpitaux Universitaires Paris Centre, Assistance Publique des Hôpitaux de Paris, 27 rue du Faubourg Saint-Jacques, 75014 Paris, France; 4 Unit of Critical Care, Respiratory Science, National Heart and Lung Institute Division, Faculty of Medicine, Imperial College London, Dovehouse Street, London SW3 6LY, UK; 5 General Intensive Care Unit, Estaing Hospital, 1 place Lucie Aubrac, 63000 Clermont-Ferrand, France; 6 Institut Cochin, INSERM U1016, CNRS UMR8104, 22 rue Méchain, 75014 Paris, France; 7 INSERM U970, Paris Cardiovascular Research Center (PARCC), European Georges Pompidou Hospital, 56 rue Leblanc, 75015 Paris, France

## Abstract

**Introduction:**

Despite experimental evidence, clinical demonstration of acute state of oxidative stress and inflammation during post-cardiac arrest syndrome is lacking. Plasma level of thioredoxin (TRX), a redox-active protein induced under conditions of oxidative stress and inflammation, is increased in various critical care conditions. We determined plasma TRX concentrations after cardiac arrest and assessed relationships with severity and outcome.

**Methods:**

Retrospective study of consecutive patients admitted to a single academic intensive care unit (ICU) for out-of-hospital cardiac arrest (between July 2006 and March 2008). Plasma levels of TRX were measured at admission, day (D) 1, 2 and 3.

**Results:**

Of 176 patients included, median TRX values measured in ICU survivors and non-survivors were, respectively: 22 ng/mL (7.8 to 77) vs. 72.4 (21.9 to 117.9) at admission (*P *< 0.001); 5.9 (3.5 to 25.5) vs. 23.2 (5.8 to 81.4) at D1 (*P *= 0.003); 10.8 (3.6 to 50.8) vs. 11.7 (4.5 to 66.4) at D2 (*P *= 0.22); and 16.7 (5.3 to 68.3) vs. 17 (4.3 to 62.9) at D3 (*P *= 0.96). Patients dying within 24 hours had significantly (*P *< 0.001) higher TRX levels (118.6 ng/mL (94.8 to 280)) than those who died after 24 hours or survived (50.8 (13.9 to 95.7) and 22 (7.8 to 77)). The area under the ROC curve to predict early death was 0.84 (0.76 to 0.91).

TRX levels on admission were significantly correlated with 'low-flow' duration (*P *= 0.003), sequential organ failure assessment (SOFA) score (*P *< 0.001), and blood lactate concentration (*P *< 0.001), but not with 'no-flow' duration or simplified acute physiology score (SAPS) II score. TRX levels and admission arterial pO^2 ^correlated negatively (r = -0.17, *P *= 0.03). Finally, cardiac arrest with cardiac etiology exhibited lower levels of TRX than in cases of extra-cardiac cause (46 ng/mL (11 to 104) vs. 68 (42 to 137), *P *= 0.01).

**Conclusions:**

Our data show for the first time that TRX levels were elevated early following cardiac arrest, suggestive of oxidative stress and inflammation occurring with this condition. Highest values were found in the most severe patients. TRX could be a useful tool for further exploration and comprehension of post-cardiac arrest syndrome.

## Introduction

Shock and intractable multi-organ failure are the main causes of death after successfully resuscitated cardiac arrest (CA) [1]. Whilst cessation and reduction of blood flow are the patent mechanisms of organ dysfunction, the pathophysiology of post-cardiac arrest syndrome is complex and remains only partially understood [2]. Ischemia/reperfusion and non-specific acute activation of the inflammatory response are thought to contribute to tissular and cellular abnormalities [3]. Uncontrolled inflammation and oxidative stress could play a central and crucial role in the onset of post-cardiac arrest syndrome. Even though supported by a large amount of experimental data, clinical investigation of these phenomena after CA is lacking. Limitations of *in vivo *analytical indexes may explain, to some extent, this knowledge gap, with issues to translate markers from bench (experimental studies) to bedside (clinical scenario).

While markers of inflammation (C-reactive protein (CRP), procalcitonin (PCT)) have assumed importance as biomarkers in critical care, their interpretations have been questioned after CA [4]. Moreover, markers of oxidative stress investigated in acute illness suggest disappointing results [5,6].

Meanwhile, translational research has highlighted the major role of thioredoxin (TRX) in physiological and pathological conditions. This ubiquitous, 12 kDa intracellular redox-active thiol protein is increased and released during inflammation and oxidative stress. Indeed, TRX, with its redox-active disulfide/dithiol site acting as a protein disulfide-reducing system, is a major intracellular redox regulatory molecule scavenging reactive oxygen species. TRX also regulates inflammation, cell signaling, growth, and apoptosis [7,8]. Intracellular TRX is released from cells on oxidative stress, leading to high extracellular levels in numerous situations relevant to critical care, including: severe burn injury [9], acute lung injury [10], and in particular, ischemia-reperfusion injury, heart disease and sepsis [11-14].

To date, neither animal nor human studies have measured plasma concentrations of TRX after CA. Thus, this study was designed to further explore the biological storm occurring after CA. We hypothesized that TRX is increased after CA, and that the magnitude of the increase is linked with clinical course. Thus, we first measured TRX levels following CA and second, determined associations between TRX levels and markers of severity of post-cardiac arrest syndrome and clinical outcomes.

## Materials and methods

### Study setting and population

All consecutive patients over 18 admitted to our 24-bed medical ICU between July 2006 and March 2008 after a successfully resuscitated CA were eligible. We retrospectively reviewed all medical records and data from our prospectively acquired ICU database, in which all CA survivors' characteristics are registered according to the Utstein style [15]. The following data were extracted prospectively for each patient: demographic data, clinical parameters, cause of CA, "no-flow" and "low-flow" period (respectively, time from collapse to basic life support and time from basic life support to return of spontaneous circulation), initial rhythm, Simplified Acute Physiology Score II (SAPS II) and Sequential Organ Failure Assessment (SOFA) scores, hypothermia management, biological parameters and ICU mortality. Post-resuscitation shock was defined as the need for vasoconstrictive drug (epinephrine or norepinephrine) infusion lasting more than 6 hours despite adequate fluid loading. Patient management was strictly standardized [2]. When employed, hypothermia was started immediately at ICU admission using external cooling by forced cold air cooling during the first 24 hours in order to obtain a target temperature between 32°C and 34°C. Sedation using adjusted doses of midazolam and morphine or fentanyl, and neuromuscular blocking agent infusion during therapeutic hypothermia, were applied. In the absence of shock or complications, sedation was interrupted at the end of the hypothermia period. Normothermia between 37°C and 37.5°C was then achieved using passive rewarming at the targeted rate of 0.3°C/hour and maintained during the next 24 hours. Patients with neither admission nor day 1 serum sample were excluded. Part of the cohort was previously studied to investigate PCT levels for the diagnosis of early onset pneumonia after CA; however, patients who died within the first 24 hours, with an infection prior to CA, or with an extra-pulmonary infection developing within 5 days following admission had been excluded [4]. The study was approved by our local Cochin University Hospital institutional review board. Written consent was waived for this study since TRX dosages did not require specific or additional blood drawing. Informed assessment was obtained from all patients or next of kin.

### Blood sampling

All TRX measurements were performed in April 2010 by the same investigator (DB) by using blood samples collected at admission, day 1 (D1), day 2 (D2) and day 3 (D3). These samples were initially centrifuged and stored at -80°C within 4 hours, as approved by our local institutional review board, as part of a serum collection. Measurements of TRX were performed in duplicate samples with a commercially available sensitive enzyme-linked immunosorbent assay (Redox Biosciences, Kyoto, Japan). Patients exhibiting hemolysis were excluded due to the high intracellular concentration of TRX, which will bias assessment [7]. Plasma levels of TRX were also determined in 30 healthy volunteers in stable condition at rest (checked for the absence of chronic or acute illness by questionnaire and medical examination). Analyses of CRP were performed with a fully automated immunoturbidimetric assay (CRPLX, Modular PP™, Roche Diagnostics, Mannheim, Germany). PCT concentrations were quantified with an immunofluorimetric assay (PCT sensitive, Kryptor™, Brahms, Berlin, Germany).

Thiol determinations (expressed as μmol/L) were based on the thiol/disulfide reaction of thiol and Ellman's reagent (5,5'-dithiobis(2-nitrobenzoic acid) DTNB). Fifty microliters of the sample mixed with 1 ml 0.1 M Tris, 10 mM EDTA pH 8.2, constituting the blank reaction, was assessed at 412 nm (UVIKON, Kontron Instruments, Milan, Italy). The addition of 40 μl 10 mM DTNB in methanol triggered the reaction and absorption at 412 nm was measured after stable colour formation (1 to 3 min). The concentrations of thiol groups were calculated using a molar extinction coefficient of 13,600 M^-1 ^cm^-1^.

Advanced oxidation protein products (AOPP) were quantified as follows: 200 μl of serum diluted 1:5 in phosphate-buffered saline was placed into each well of a 96-well microtiter plate and added 20 μl of acetic acid to each well. For the standards, we added 10 μl of 1.16 M potassium iodide (Sigma-Aldrich, St Louis, MO, USA) to 200 μl of chloramine-T solution (0 to 100 μmol/l) (Sigma-Aldrich, St Louis, MO, USA) in a well and then added 20 μl of acetic acid. The absorbance of the reaction mixture was immediately read at 340 nm against a blank consisting of 200 μl of phosphate-buffered saline, 10 μl of 1.16 M potassium iodide, and 20 μl of acetic acid. AOPP concentrations are expressed as micromoles/liter of chloramine-T equivalents.

### Statistical analysis

Continuous variables were expressed as medians (with interquartile range) and qualitative variables were reported as count and proportions, unless specified otherwise. Statistical analysis compared ICU survivors and non-survivor patients with nonparametric tests, as appropriate: continuous variables with the Wilcoxon rank sum test; categorical variables with the χ^2 ^test. Statistical significance was defined as *P *< 0.05. Correlation was performed with the Spearman test. Receiver-operated characteristic (ROC) curves were performed to assess the ability of TRX concentrations to predict ICU death. Results were expressed with area under the curve (AUC) and 95% confidence interval. Analyses were performed with Stata 7.0 software (StataCorp., College Station, TX, USA).

## Results

During the 20-month study period, 245 patients were admitted for a successfully resuscitated CA. After excluding 59 patients with neither sample at admission nor D1 and 10 patients with hemolytic blood samples, we enrolled 176 consecutive patients in the final analysis.

Cohort had a median age of 60 years (48 to 73) and 116 patients were men. Characteristics of CA were: 'no-flow' duration 5 (0 to 10) min, 'low-flow' duration 15 (8 to 25) min, initial shockable rhythm *n *= 71 (41%), and cardiac etiology *n *= 93 (53%). Non-cardiac causes were respiratory (*n *= 39), neurological (*n *= 9) and miscellaneous (*n *= 35). Severity of the population was highlighted by SAPS II score of 68 (60 to 81) and admission SOFA score of 9 (6 to 12). Post-resuscitation shock occurred in 131 patients (74%); 152 patients (89%) were treated with therapeutic hypothermia. ICU mortality rate reached 61% (107 patients), whereas most of the survivors were Cerebral Performance Category 1 or 2 (*n *= 64). Table 1 reports the main characteristics of ICU survivors and non-survivors.

**Table 1:** Baseline admission characteristics and outcome of ICU survivors and non-survivors.

	All patients (*n *= 176)	Survivors (*n *= 69)	Non-survivors (*n *= 107)	*P *
**Age (year)**	60 (48-73)	57 (46-68)	60 (49-74)	0.18
**No-flow duration (min)**	5 (0-10)	2 (0-5)	5 (1-10)	0.004
**Low-flow duration (min)**	15 (8-25)	10 (5-16)	20 (10-26)	< 0.001
**Shockable rhythm**	71 (41)	47 (68)	24 (22)	< 0.001
**Cardiac etiology**	93 (53)	44 (68)	47 (44)	0.02
**SAPS II score**	68 (60-81)	68 (61-82)	67 (58-81)	0.55
**Admission SOFA score**	9 (6-12)	7 (5-10)	11 (8-13)	< 0.001
**Admission temperature (°C)**	35.4 (34.7-36.6)	35.8 (34.8-36.5)	35.3 (34.7-36.9)	0.65
**Therapeutic hypothermia**	152 (89)	65 (94)	87 (81)	0.02
**Post-resuscitation shock**	131 (74)	49 (71)	82 (76)	0.4

Data are expressed as median (with interquartile range) or absolute value (%). Statistical analysis is performed between survivors and non-survivors. SAPS II, Simplified Acute Physiology Score II; SOFA, Sequential Organ Failure Assessment.

**Figure 1:**
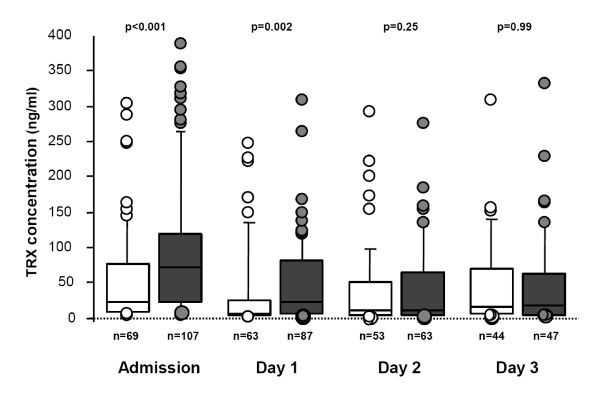
**Serum thioredoxin (TRX) levels on admission, then 1, 2 and 3 days after cardiac arrest, according to the ICU survival**. White boxes represent ICU survivors, grey boxes represent non-survivor patients. The median is shown by the horizontal line within the box. The values between the lower and upper quartiles (25^th ^to 75^th ^centiles) are within the box.

**Figure 2:**
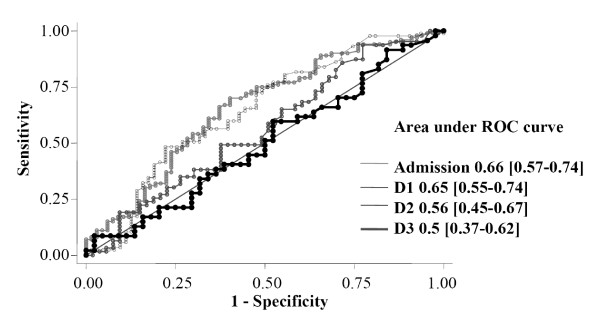
**Receiver-operated characteristic (ROC) curves comparing the ability of thioredoxin (TRX) concentrations to predict ICU death at admission, day 1, day 2 and day 3**.

TRX concentration was 10.7 ng/mL (9.1 to 20.9) in healthy volunteers (14 male, age 49 (39 to 54)). In our patient cohort, median serum TRX values in ICU survivors and non-survivors were respectively (Figure 1): 22 ng/mL (7.8 to 77) vs. 72.4 (21.9 to 117.9) at admission (*P *< 0.001), 5.9 (3.5 to 25.5) vs. 23.2 (5.8 to 81.4) at D1 (*P *= 0.003), 10.8 (3.6 to 50.8) vs. 11.7 (4.5 to 66.4) at D2 (*P *= 0.22), and 16.7 (5.3 to 68.3) vs. 17 (4.3 to 62.9) at D3 (*P *= 0.96). The areas under the ROC curves of TRX that discriminates survivors and non-survivors were: 0.66 (0.57 to 0.74) at admission, 0.65 (0.55 to 0.74) at D1, 0.56 (0.45 to 0.67) at D2 and 0.5 (0.38 to 0.62) at D3 (Figure 2).

**Figure 3:**
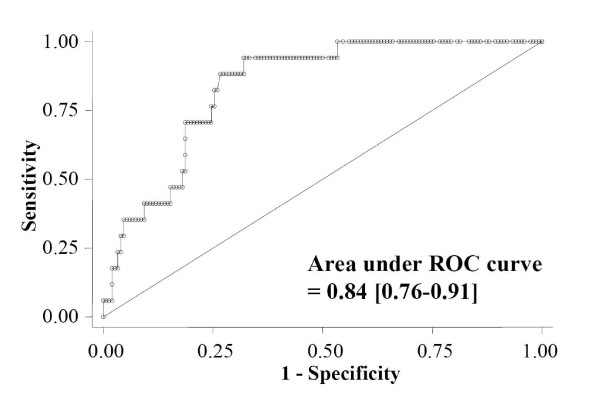
**Receiver-operated characteristic (ROC) curve determining the ability of thioredoxin (TRX) concentration to predict death within 24 hours**.

When timing of death was considered, patients dying within 24 hours (*n *= 17) had higher admission TRX levels (118.6 ng/mL (94.8 to 280)) compared with cases of late death or survival (respectively, 50.8 (13.9 to 95.7) and 22 (7.8 to 77), *P *< 0.001); area under ROC curve to predict early death was 0.84 (0.76 to 0.91) (Figure 3). Refractory shock was the cause of 88% of these early deaths.

Admission TRX correlated significantly with 'low-flow' duration (r = 0.24, *P *= 0.003), SOFA score (r = 0.27, *P *< 0.001), and admission arterial lactate concentration (r = 0.38, *P *< 0.001), but was not associated with 'no-flow' duration (r = 0.07, *P *= 0.39) or SAPS II score (r = 0.04, *P *= 0.6). TRX levels and admission arterial pO^2 ^correlated, negatively (r = -0.17, *P *= 0.03).

Finally, patients experiencing CA due to a cardiac etiology exhibited lower levels of TRX at admission than in cases of extra-cardiac cause (46 ng/mL (11 to 104) vs. 68 (42 to 137), *P *= 0.01); similarly, patients with shockable rhythm had lower admission TRX concentrations (16.5 (6.5 to 73.7) vs. 74 (27 to 132) than in cases of non-shockable rhythm).

Routinely available inflammation biomarkers, CRP and PCT, were also measured and thiol group formation and AOPP quantified. Non-survivors exhibited higher CRP levels at admission and at D1, whereas their PCT concentrations were higher from admission to D3. Conversely, AOPP and thiol concentrations did not differ irrespective of outcome (Table 2).

**Table 2:** Plasma concentrations of thiol, advanced oxidation protein product (AOPP), C-reactive protein (CRP) and procalcitonin (PCT) according to the outcome.

	Survival *n *= 69	Early death (< 24 h) *n *= 17	Late death (> 24 h) *n *= 90	*P*
**Thiols (μmol/L)**				
Admission	275 (223-316)	251 (193-358)	247 (193-299)	0.31
Day 1	310 (239-358)		299 (219-360)	0.97
Day 2	272 (221-319)		267 (228-331)	0.64
Day 3	240 (191-273)		244 (210-292)	0.23

**AOPP (μmol/L)**				
Admission	36.8 (22.2-64.7)	42 (25.3-78.6)	40.4 (21.7-59.5)	0.87
Day 1	27.9 (14.7-54.1)		37 (23.4-60.4)	0.11
Day 2	32.4 (19.9-65.8)		36.5 (25.8-62.1)	0.43
Day 3	28.4 (22.3-48)		28.9 (28.9-58.6)	0.98

**CRP (mg/mL)**				
Admission	2.15 (1-6.5)	4.6 (1-28.9)	3.95 (1.3-32.7)	0.05
Day 1	42.1 (18-1-79.6)		66.2 (19.9-135.8)	0.02
Day 2	137.4 (94.9-180)		158 (84-198)	0.34
Day 3	176 (102-199)		170 (79.9-217)	0.94

**PCT (ng/mL)**				
Admission	0.18 (0.1-0.67)	0.55 (0.23-2.2)	0.66 (0.18-3.74)	0.0004
Day 1	1 (0.25-6.04)		6.38 (1.18-29.7)	0.0001
Day 2	0.82 (0.22-4.83)		4.54 (1.28-29.2)	0.0001
Day 3	0.96 (0.28-3.45)		5.8 (1.08-19.6)	0.0002

Data are expressed as median, with interquartile range. Statistical analysis is performed between survivors and non-survivors.

## Discussion

In a large cohort of CA patients, we assessed the levels of plasma TRX and found that very high levels occurred after CA, with an early peak and subsequent decrease over 3 days; highest levels were associated with worst outcome. To our knowledge, this is the first study evaluating the potential usefulness of TRX determination for assessment of both pathophysiology and severity after CA.

The pathophysiology of post-cardiac arrest syndrome is dominated by a global ischemia-reperfusion phenomenon and non-specific activation of the systemic inflammatory response [2]. During the 'no-flow' phase of CA, reduced oxygen supply leads quickly to cellular damage. Reperfusion ('low-flow' phase of CA), generates a burst of radical oxygen species production [3,16-18]. A number of animal studies have explored the role of radical oxygen species in organ damage after CA [19,20]. Other studies revealed that oxidative stress increased quickly after CA, peaked during early reperfusion and subsided rapidly, suggesting that oxidant injury contributes widely to the lesions observed after CA [21,22]. In particular, the oxidative stress status during CA may inactivate myocardial enzymes and thereby cause ischemic derangements of myocardial metabolism. Similar features in humans were only recently shown in a study in which plasma of out-of-hospital CA survivors induced acute and major endothelial toxicity, attributable to an acute pro-oxidant state occurring within the cells, as shown by a significant decrease of the main antioxidant defences. Another striking finding was that plasma toxicity lasting for more than 3 days after CA [23].

With respect to the inflammatory response associated with CA, a wide panel of proteins and biomarkers investigated in several animal models and human cohorts suggests that a major inflammatory syndrome occurs after CA [2,24,25]. Consequently, post-cardiac arrest syndrome was defined as a 'sepsis-like syndrome', with clinical, biochemical and hematological features that are very similar to those observed during severe sepsis, and increased levels of pro- and anti-inflammatory cytokines comparable to the variations described during septic shock [24,26]. Moreover, disseminated vascular endothelial damage also suggests that ischemia-reperfusion associated with CA evolves toward systemic inflammation with overproduction of cytokines, complement activation, synthesis of arachidonic acid metabolites, expression of leukocyte adhesion molecules and activation and chemotaxis of polymorphonuclear neutrophils contributing to the inflammatory response [2].

TRX concentrations in healthy volunteers were similar in our study to those previously reported by others (15 to 25 ng/mL). Also, increased levels of TRX of 36.1 ng/mL in patients with acute lung injury [10] and up to 161.6 ng/mL in patients with sepsis [14] are similar to the levels and magnitude of increase we found in patients following CA. TRX was also found to be elevated after cardiopulmonary bypass or heart failure, two clinical situations that combine great inflammation and circulatory disturbances [27,28]. Moreover, as has been previously reported for patients with sepsis [14] or meningococcal septic shock [11], we found that TRX levels were significantly higher in non-survivors than in survivors, even if ability to predict ICU death was not robust. In addition, patients dying within 24 hours exhibited the highest levels of TRX, with admission concentration carrying a very good ability to predict early death.

Our findings suggest that patients suffer from major oxidative stress and inflammation during post-cardiac arrest syndrome, which cannot be counteracted by increased TRX production. Others have suggested that prolonged oxidative stress in patients with coronary risk factors wastes the serum antioxidant pool such as vitamin C and that serum TRX is recruited to compensate [29]. That our data show highest levels associated with worse outcome following CA is perhaps suggestive of the severity of oxidative stress associated with the condition. These findings confirm and broaden the data of post-cardiac arrest syndrome pathophysiology, supporting the hypothesis that oxidative stress and inflammatory insults are much more marked in the most severe patients and contribute largely to the high initial mortality. Likewise, when focusing on the cause of CA, cardiac etiologies had lower TRX levels. This is in line with the overall better prognosis of CA of coronary origin [30].

When focusing on disease severity, TRX concentrations were correlated to admission arterial lactate levels, a biological parameter that is constantly associated with unfavorable outcome [31]. We also found a strong association between low-flow duration and TRX levels, whereas this was not observed with the no-flow duration. This illustrates the pathophysiology of the ischemia reperfusion injury, with a major reactive oxygen species production during the reperfusion phase [16,17,32]. This is also consistent with the well-known observation that the severity of the post-cardiac arrest syndrome is much more driven by the low-flow duration [33]. Finally, the absence of correlation with SAPS II, by contrast with association with SOFA score, could be explained by the fact that SOFA score describes strictly organ failures [34]. Conversely, SAPS II score takes into account not only medical condition, but also underlying comorbidities [35].

Perhaps, surprisingly, we observed that TRX levels decreased within of short range of time. As inflammatory and pro-oxidant states are known to persist after CA, this finding might suggest that TRX half-life is relatively short, or that release is minimized after the initial insult, or that other serum antioxidants have been replenished. This contrasts with sustained levels of other biomarkers such as PCT, which remain elevated 3 days after CA [4]. The difference in kinetic profile is intriguing and warrants further investigation.

The negative correlation between plasma TRX levels and arterial pO^2 ^at admission that we found in our study advocates against a role of hyperoxia in the onset of oxidative stress. There is a great controversy surrounding hyperoxia after CA resuscitation, with some experimental data suggesting that hyperoxia might increase oxidative stress. While animal models were inconclusive [36], a human study suggested that hyperoxia was independently associated with in-hospital mortality, as compared with hypoxemia or normoxia [37]. However, the latter finding was refuted by both a study, in which this association did not appear in a large cohort, with adjustment on severity scores [36] and also our findings in the current work. Nevertheless, further studies are required to elucidate this hot topic [38,39].

TRX reflects both inflammation and oxidative stress, two major determinants of severity of post-cardiac arrest syndrome and could be considered as a potential marker of the global insult. However, the clinical utility of TRX has to be further established [40], as does the mechanistic explanation linking increased TRX levels with the biological disorders occurring after CA and post-cardiac arrest syndrome. TRX is elevated on admission, whereas vasoplegia and myocardial dysfunction typically begin a few hours after CA. In the future, utilizing biomarkers of inflammation and oxidative stress might allow tailoring therapeutic interventions that modulate inflammation or oxidative stress such as high volume hemofiltration [41] or steroids administration [42].

Besides its role of biomarker, TRX could be a future therapeutic target. Hofer *et al. *demonstrated, in an experimental model of cecal ligature and puncture, that neutralization of endogenous TRX was deleterious for septic mice survival, whereas treatment with recombinant TRX markedly enhanced their survival [14]. To date, no human data of such protective effects of TRX is available. However, major hope arose in the field of resuscitation after the use of coenzyme Q10, a mitochondrial enzyme playing a key role in antioxidant defense. In 49 patients experiencing CA, exogenous administration of this antioxidant improved both survival and neurological outcome [43]. Further investigations are required to determine the place of TRX modulation in the armamentarium of critical care therapeutics.

Although these data suggest that TRX could reflect a response to inflammation and oxidative stress, the functional consequences of high levels of TRX are not completely understood. The potential biological significance of TRX upregulation can be inferred from experimental studies, which support a role as an antioxidant and anti-inflammatory protein, through modulation of both heme oxygenase-1 and NADPH oxidase-mediated generation of superoxide anion. Extracellular TRX has been reported to reduce interleukin 1-beta expression by monocyte-macrophages in inflammatory conditions [7]. In addition, circulating TRX suppresses neutrophil chemotaxis [44]. More recently, it has been suggested that the anti-inflammatory mechanisms of TRX could be mediated, at least in part, by migration inhibitory factor (MIF) downregulation [45]. However, in septic conditions, TRX and MIF plasma levels showed a strong correlation, suggesting that pro- and anti-inflammatory agents are balanced to maintain homeostasis [46].

In the present study, we confirm the limited interest of AOPP measurement and thiol content determination in acute setting, at least after CA, despite their place in experimental conditions [5,6]. On the contrary, high CRP and PCT levels are consistent with previous findings suggesting the overwhelming impact of systemic inflammation [4]. Overall, these results enhance the value of TRX after CA.

Despite the large number of post-CA patients enrolled, some limitations of this study need consideration. First, we considered a retrospective single-institution cohort. However, all analyzed data were prospectively collected, and medical management was homogeneous. Second, this is a merely observational study, allowing only association rather than causation conclusions. Third, the utility of TRX to predict death was evaluated as a single parameter, without combination of TRX level to clinical or biological data that may have improved the overall prognosis value. Fourth, measurement of other oxidative stress parameters, like MIF or manganese superoxide dismutase (MnSOD), or main inflammatory cytokines would have provided further mechanistic tracks. Similarly, determination of the TRX interacting protein (TXNIP) could give valuable data to help explain the TRX kinetics after CA. However, TXNIP is an intracellular protein that cannot be routinely measured in serum samples. Fifth, CA has the particularity of exhibiting two main causes of ICU death, that is, shock and neurological damage with subsequent care withdrawal. As inflammation and oxidative stress are expected to be greater in case of shock, subanalysis focused on this last group. Finally, the vast majority of patients underwent therapeutic hypothermia. If the relationship between inflammatory cytokines or biomarkers and effect of therapeutic hypothermia is controversial [4,25], the influence of hypothermia on TRX is as yet, unknown. As the vast majority of our patients were treated by hypothermia, we could not perform a meaningful analysis of the impact of temperature on TRX levels. Similarly, there is no data about clearance of TRX during renal replacement therapy, which is widely applied in post-cardiac arrest survivors. To note, we report a high proportion of patients experiencing post-resuscitation shock. This is related to the study design, including all consecutive patients and in particular those dying within 24 hours. These most severe patients are generally excluded from clinical studies. Nonetheless, it is likely to be the group of patients of the most significant clinical interest.

## Conclusions

In patients successfully resuscitated from CA, serum TRX levels measured during the first 3 days were higher in ICU non-survivors, and were dramatically greater in patients dying early from circulatory failure. Besides assessing severity and outcome, such a biomarker offers interesting perspectives in the comprehension and management of post-cardiac arrest syndrome.

## Key messages

• Thioredoxin (TRX), a surrogate global marker of inflammation and oxidative stress, was largely increased during post-cardiac arrest syndrome.

• The highest values were found in the most severe patients.

• Cardiac arrest with cardiac etiology exhibited lower levels of TRX than in cases of extra-cardiac cause.

• Admission TRX levels were significantly correlated with other pertinent severity markers, like 'low-flow' duration, SOFA score, and arterial lactate concentration.

• No correlation was found between TRX levels and admission arterial pO^2^, arguing against a potential role of hyperoxia after cardiac arrest.

## Abbreviations

AOPP: advanced oxidation protein products; AUC: area under the curve; CA: cardiac arrest; CRP: C-reactive protein; ICU: intensive care unit; kDa: kiloDaltons; MIF: migration inhibitory factor; MnSOD: manganese superoxide dismutase; PCT: procalcitonin; ROC: receiver-operated characteristic; SAPS II: Simplified Acute Physiology Score II; SOFA: Sequential Organ Failure Assessment; TRX: thioredoxin; TXNIP: TRX interacting protein.

## Competing interests

The authors declare that they have no competing interests.

## Authors' contributions

NMo, DB and AC designed the study. NMo, VL and SP extracted the data. DB performed the biochemical analysis. VL performed the statistical analysis. ABG, SP, NMa, JC, FP, JDC and JPM contributed to the conduct of study and data analysis. NMo and AC wrote the manuscript. All the authors read and approved the final version of the manuscript.
